# Predictability of intelligence and age from structural connectomes

**DOI:** 10.1371/journal.pone.0301599

**Published:** 2024-04-01

**Authors:** Sebastian J. Kopetzky, Yong Li, Marcus Kaiser, Markus Butz-Ostendorf, for the Alzheimer’s Disease Neuroimaging Initiative

**Affiliations:** 1 Labvantage—Biomax GmbH, Planegg, Germany; 2 School of Life Sciences, Technical University of Munich, Freising, Germany; 3 Precision Imaging Beacon, School of Medicine, University of Nottingham, Nottingham, United Kingdom; 4 Department of Functional Neurosurgery, Rui Jin Hospital, Shanghai Jiao Tong University, Shanghai, China; 5 Laboratory for Parallel Programming, Department of Computer Science, Technical University of Darmstadt, Darmstadt, Germany; Georgia Institute of Technology, UNITED STATES

## Abstract

In this study, structural images of 1048 healthy subjects from the Human Connectome Project Young Adult study and 94 from ADNI-3 study were processed by an in-house tractography pipeline and analyzed together with pre-processed data of the same subjects from braingraph.org. Whole brain structural connectome features were used to build a simple correlation-based regression machine learning model to predict intelligence and age of healthy subjects. Our results showed that different forms of intelligence as well as age are predictable to a certain degree from diffusion tensor imaging detecting anatomical fiber tracts in the living human brain. Though we did not identify significant differences in the prediction capability for the investigated features depending on the imaging feature extraction method, we did find that crystallized intelligence was consistently better predictable than fluid intelligence from structural connectivity data through all datasets. Our findings suggest a practical and scalable processing and analysis framework to explore broader research topics employing brain MR imaging.

## Introduction

The connectome—the entire map of neural connections—uniquely represents every subject’s gender, age and intelligence like a fingerprint [1]. Intelligence is known to be affected by, e.g., topological properties of brain networks such as characteristic path length and global network efficiency, respectively [2, 3]. The association between a lower characteristic path length and IQ has also been described for resting state functional MR imaging (rs-fMRI) networks [4]. Predicting not only gender [5, 6] and age [7, 8] but also different forms of intelligence [5, 6, 9–11] in individual subjects made significant progress by using rs-fMRI. However, far less is known to what degree the underlying structural connectome, the backbone of the functional interactions, is also predictive of age and intelligence in cognitively normal adults. Several studies have tested the predictability of brain age using more advanced machine learning models. For example, Lin et al. predicted older individuals’ age using artificial neural networks [12] and Taoudi-Benchekroun et al. used deep neural networks and random forests to predict the age of infants [13].

Previous studies suggest a distinct nature with normal aging between crystallized and fluid intelligence [14]. Fluid intelligence shows one’s ability to acquire new knowledge and is reflected in problem-solving and adaptation to unknown environments therefore it examines cognitive tasks such as cognitive flexibility, working memory, and information processing speed, while crystallized intelligence more reflects experience-based knowledge and the ability to access it and is e.g. measured by vocabulary and decoding tasks [15–18]. Shokri-Kojori et al. [19] compared age-related variance between younger and older adults (100 subjects) for gray matter (GM) and white matter (WM) tissue-specific age scores. They found that the WM age score accounted for significantly more variance in chronological age and was negatively associated with crystalized intelligence in older adults. Góngora et al. [20] reconstructed 10 tracts by deterministic tractography in 83 healthy individuals from the Cuban Human Brain Mapping Project. Their results showed predictive effects of the forceps minor tract on crystallized intelligence and of the superior longitudinal fasciculus on fluid intelligence.

In this study, we explored how well different intelligence measures and age of cognitively normal adult subjects can be predicted from the structural connectome as quantified by diffusion-weighted imaging (DWI). Specifically, we processed DWI data of HCP young adult (1048 subjects) and ADNI-3 (94 cognitive normal elderly subjects) datasets to reconstruct whole brain structural connectome features by our in-house tool, NICARA. Then, we used NICARA extracted features to apply the correlation-based regression (CBR) machine learning method [21] to predict age as well as total, fluid and crystallized intelligence. A similar approach was also suggested by Shen et al. [22]. To further explore the predictive capabilityof the CBR ML model and allow for additional statistical comparisons, we also included the structural connectome features of the HCP dataset preprocessed by braingraph.org available with different parcellations.

## Materials and methods

### Ethics statement

According to national law and institutional rules research involving the analysis of existing data, where the data is either already publicly available or will be analyzed such that individual subjects cannot be identified is exempt from IRB oversight.

### Datasets

We investigated the prediction capability for different intelligence measures and age of two different DTI pipelines (NICARA [23] and braingraph.org [24]) based on structural connectivity data of 1048 subjects from the Human Connectome Project (HCP) young adult study [25]. Investigated features were age, total intelligence, fluid intelligence, and crystallized intelligence (Table 1). The intelligence measures were unadjusted cognitive function composite score, fluid cognition composite score, and crystallized cognition composite score, respectively, based on the NIH toolbox [15].

**Table 1:** Feature distributions of the HCP subjects.

	Min	Max	Range (Max—Min)	Mean	Standard dev.
Age [years]	22	37	15	28.75	3.68
Intelligence, total	88.50	153.36	64.86	122.26	14.46
Intelligence, fluid	86.68	145.17	58.49	115.39	11.54
Intelligence, crystallized	90.95	153.95	63.00	117.92	9.81

Distributions of age as well as total, fluid and crystallized intelligence (measured as unadjusted cognitive function composite score, fluid cognition composite score, and crystallized cognition composite score, respectively) for the 1048 subjects are shown.

The braingraph.org database was constructed by deterministic ROI-based fiber tracking (10 × averaged) of 1064 healthy subjects from the HCP Young Adult dataset. It was available with five different sets of ROIs (86, 129, 234, 463 and 1015 ROIs) and therefore also provides a good opportunity to investigate the influence of the parcellation method on the prediction outcome.

The full HCP dataset consisted of 1065 subjects, and we excluded subjects for whom no data was available from braingraph.org (2 subjects) and for whom not all investigated features were available (15 subjects). After this, 1048 subjects (S1 Table) remained for the analysis (484 male, 564 female).

**Fig 1:**
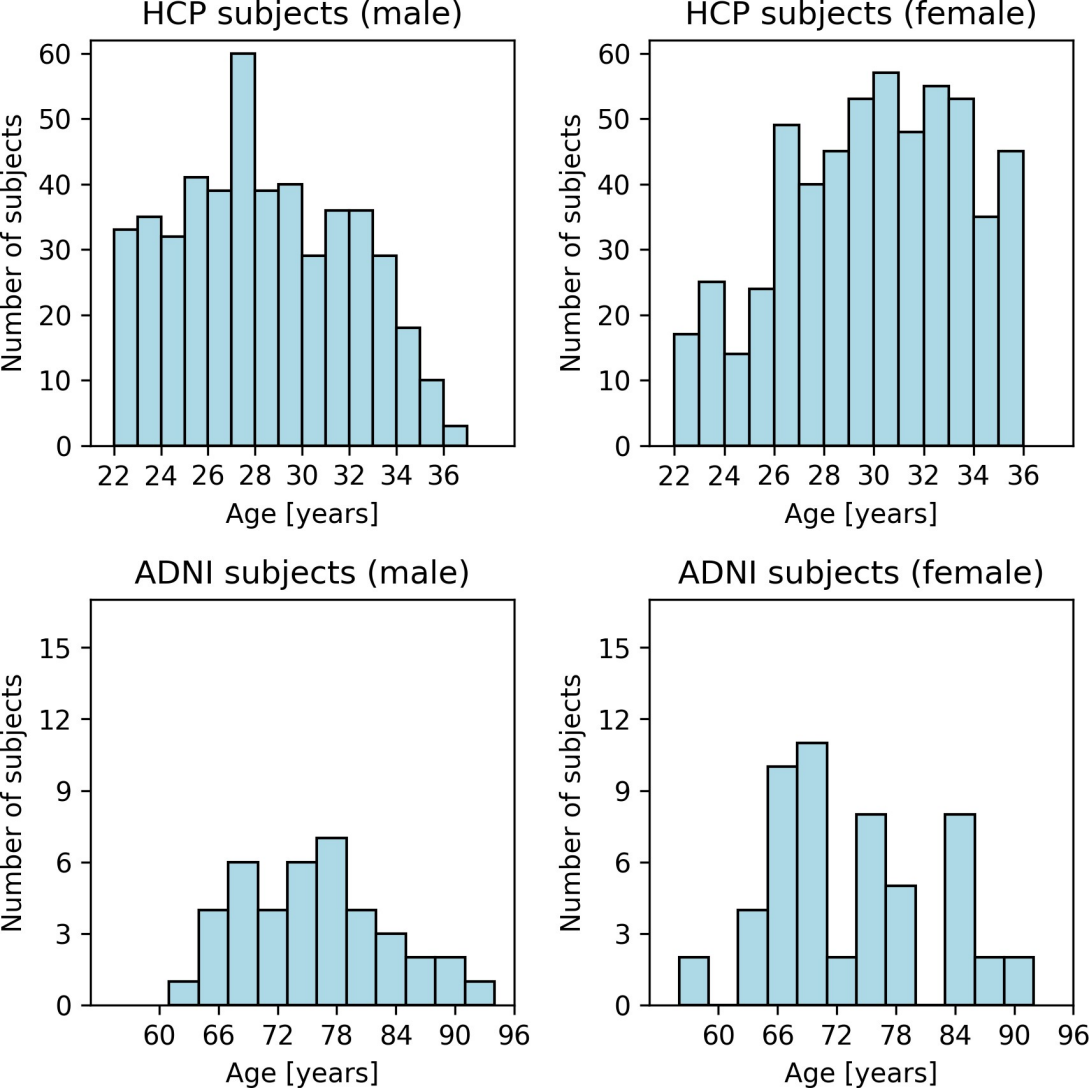
Gender-specific age distribution of the HCP and ADNI subjects.

Since the age range of subjects from the HCP Young Adult cohort is relatively small (22–37 years with a mean of 28.75 years and a standard deviation of 3.68 years; Table 1), we decided to additionally include a second cohort with adult and aged healthy subjects from the ADNI-3 study of the Alzheimer’s Disease Neuroimaging Initiative [26]. We used this second cohort (S2 Table) with 94 control subjects (40 male, 54 female) with an age range from 56.5 to 91.5 years (mean: 74.43 years, standard deviation: 7.86 years; Table 2) to confirm age predictability from connectomes extracted by our in-house pipeline. Fig 1 shows the age distribution of male and female subjects from both studies.

**Table 2:** Age distribution of the ADNI subjects.

	Min	Max	Range (Max—Min)	Mean	Standard dev.
Age [years]	56.5	91.5	55.0	74.43	7.86

Age distribution of 94 cognitively normal aged subjects from the ADNI study.

### Data processing

We processed structural connectomes of 1048 subjects from the HCP young adult study as well as the 94 ADNI subjects. In-house processing pipelines used 379 ROIs with 360 cortical ROIs (HCP-MMP1.0, Human Connectome Project Multi-Modal Parcellation version 1.0 atlas [27] and 19 subcortical ROIs (Harvard-Oxford subcortical atlas [28–30]), and 50 million streamlines for the probabilistic whole brain tractography. As comparison datasets to better assess the influence of different parcellations, we downloaded preprocessed connectivity data derived from the same HCP subjects available from the braingraph.org database [24] that have been obtained with different processing routines. braingraph.org datasets were available with five different numbers of ROIs (86, 129, 234, 463 and 1015 ROIs).

In our in-house processing pipeline, at first the T1w images get defaced using SPM12 (https://www.fil.ion.ucl.ac.uk/spm/). The T1w and DTI images were co-registered, and the images in native space were transformed into the MNI152 space by normalization. Tissue-based segmentation was performed using the CAT12 toolbox [31]. Defaced T1w images got skull-stripped using the adaptive probability region-growing (APRG) approach and used as input for FreeSurfer [32], which was used to apply cortical & subcortical parcellation in order to project the HCP MMP 1.0 and Harvard-Oxford subcortical atlas to native space via “fsaverage”. Noise and distortion correction methods were applied to the DWI images using mrtrix3 [33], FSL [34], and ANT (Advanced Normalization Tools) [35]. Anatomically Constrained Tractography (ACT) was applied using mrtrix3 with 50 million streamlines yielding the 379 x 379 connectivity matrices used for the analysis. All processing steps were assembled to a stand-alone pipeline optimized for automated execution (referred to as NICARA or in-house pipeline) and run by our proprietary neuroimaging solution NICARA Version 2.0, Labvantage—Biomax GmbH, Planegg Germany (https://nicara.eu).

### Feature prediction

In order to predict features based on the structural connectivity matrices, we applied the correlation-based regression algorithm proposed by Han et al. [21].

Given *n* subjects and *m* edges (depending on the brain parcellation), we first obtained a correlation vector *R* of size *m* from the correlation between the matrix *A* of unnormalized connection strengths of size *n x m* (containing the vectorized connectivity matrices per subject as rows) and the attribute of interest (Eq 1).


$$R_{i}=r(A_{i},b)$$ (1)

The correlation vector *R* contains the Pearson’s correlation coefficients *r* (Eq 2) between A_i_ (with A_i_ being the *i*th column of matrix A) and *b*, a vector of length *n* containing the values of the attribute of interest (e.g. age) for each subject.


$$r_{A_{i},b}=\frac{\sum \left(A_{i}-m_{A_{i}})(b-m_{b}\right)}{\sqrt{\sum {\left(A_{i}-m_{A_{i}}\right)}^{2}\sum {\left(b-m_{b}\right)}^{2}}}$$ (2)

Subsequently, we calculated the vector of predictor values S of size *n* for the regression analysis by summing up each subject’s connection strengths weighted by the respective correlation coefficient of the edge (Eq 3).


$$S_{j}=\sum_{x=0}^{m-1}A_{x,j}\cdot R_{x}$$ (3)

The predictor value S_j_ for subject *j* is the scalar product of the vectors *A*_j_ and *R*_x_.

Based on the predictor values we fitted a simple linear least-squares regression model that was then used for predicting the feature value of interest. To assess and compare the prediction quality, a 10-fold cross-validation was performed for each dataset. *R* was calculated from the training data only and the Pearson’s *r between predicted and actual values* as well as the mean absolute error (Eq 4) and range-normalized mean absolute error were calculated from the ten iterations of the cross-validation.


$$MAE=\frac{\sum_{i=1}^{k}\left|g_{i}-h_{i}\right|}{k}$$ (4)

The mean absolute error (MAE) is the norm of the difference of predicted values g and the real values h. The range-normalized mean absolute error (NMAE) can be obtained by dividing the MAE by the difference of the maximal and minimal value of the data and therefore allows for a better comparability between different datasets (unpaired data).

## Statistical analysis

Statistical analysis was performed based on the paired absolute errors of subjects obtained from the cross-validation using the Wilcoxon signed-rank test. Comparisons were done between the different datasets of the same feature to determine differences in the as well as within each dataset between crystallized and fluid intelligence. We applied the Benjamini-Hochberg multiple testing correction with a false discovery rate (FDR) of 0.05 to the p-values of each analysis.

## Results

We applied the correlation-based regression algorithm (S1 File) to predict age for different HCP datasets comprising 1048 subjects (NICARA, braingraph.org datasets) and a subset of the ADNI study comprising 94 subjects, as well as different intelligence measures in case of the HCP datasets. Group comparisons were performed based on the mean absolute error (Eq 4) as well as the Pearson correlation coefficient between the actual and predicted values based on a regression analysis of the total data as well as gender-specific subgroups. Then we applied the Wilcoxon signed-rank test for age, total, crystallized and fluid intelligence to identify differences in the paired absolute errors over the different pipeline conditions. Finally, Wilcoxon signed-rank tests of the paired absolute errors revealed significant differences between crystallized and fluid intelligence in all comparisons.

### Age prediction

For the HCP datasets, the maximal observed Pearson correlation coefficients between the features and age per dataset ranged from 0.105 for the 129 node braingraph dataset to 0.227 for the in-house pipeline (S3 Table), while the minimal Pearson coefficients ranged from -0.104 for the in-house pipeline to -0.168 for the 86 and 129 node braingraph datasets (S4 Table). The maximum for the ADNI dataset was r = 0.619 and the minimum r = -0.550, therefore stronger compared to the values observed in the HCP datasets.

**Fig 2:**
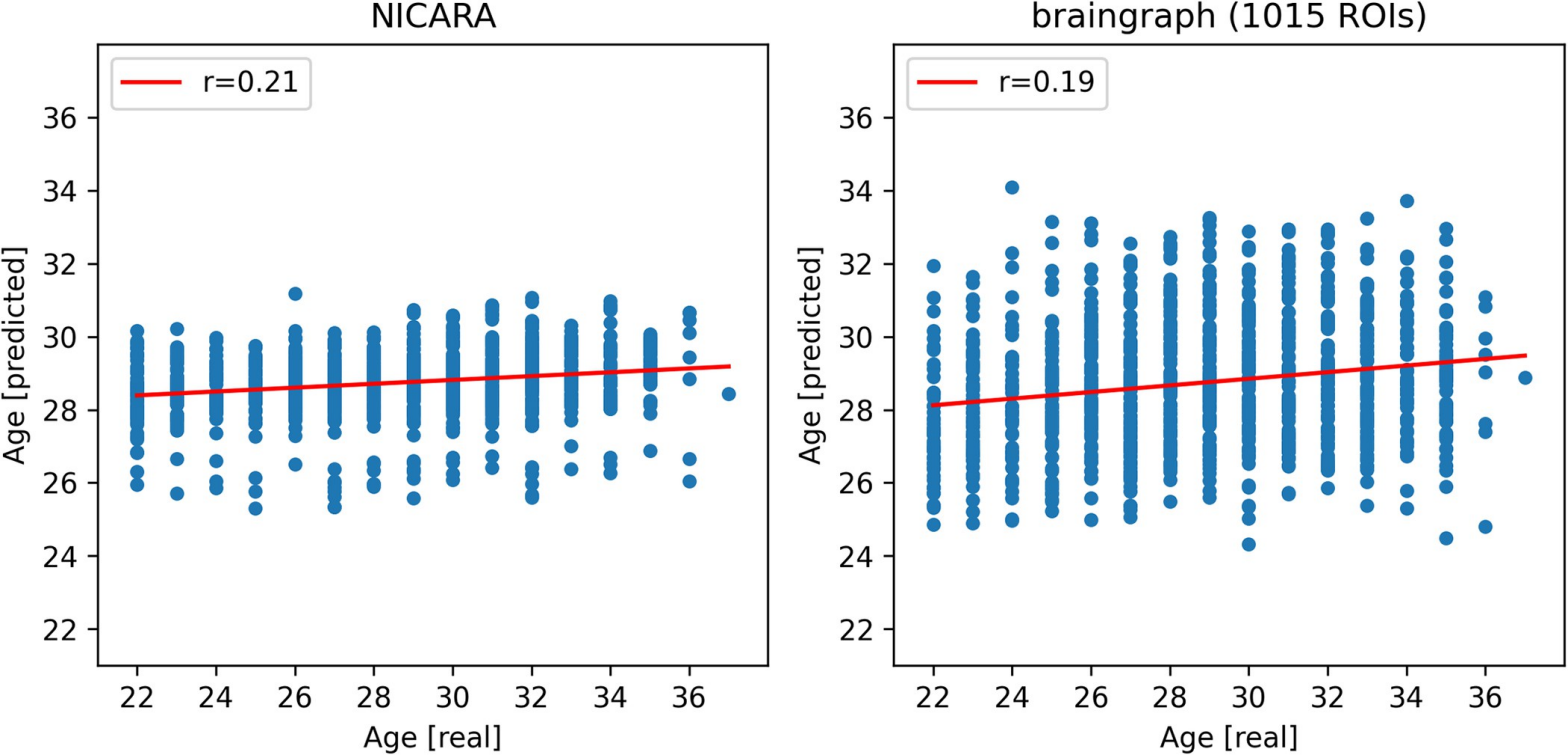
Prediction results for the HCP dataset (age) for NICARA and the 1015 ROI dataset from braingraph.org. The x-axis shows the actual age in years and the y-axis the predicted age in years during the cross-validation.

Our in-house pipeline achieved the highest Pearson correlation between actual and predicted age values (r = 0.21) and the lowest mean absolute error (MAE = 3.02) out of the datasets (Table 3; Fig 2). Within the braingraph datasets, the MAE slightly increased with the number of ROIs (3.02 to 3.13). Based on the Wilcoxon signed-rank test for the paired absolute errors of the full datasets, the difference between the NICARA dataset and the following braingraph datasets was significant before a multiple testing correction: braingraph 129 ROIs (p = 0.0251), braingraph 234 ROIs (p = 0.0245), braingraph 463 ROIs (p = 0.0127) and braingraph 1015 ROIs (p = 0.0085). Within the braingraph datasets, the following were significant: 86 vs 1015 ROIs (p = 0.0350), 234 vs 463 ROIs (p = 0.0478) and 234 vs 1015 ROIs (p = 0.0251). However, after applying the Benjamini-Hochberg multiple testing correction (FDR = 0.05), none of the p-values remained significant. In the gender-specific subgroups, the following two datasets showed a significant p-value for males before but not after multiple testing correction: 86 vs 129 ROIs (p = 0.035) and 86 vs 234 ROIs (p = 0.006).

**Table 3:** Prediction outcome using the correlation-based regression method for age.

	Pearson‘s r	MAE	NMAE
NICARA	**T: 0.21 (p = 2.72e-12)**	**T: 3.02 (±1.95)**	**T: 0.2012**
379 ROIs	M: 0.14 (p = 0.0018)	M: 3.04 (±1.97)	M: 0.2024
	**F: 0.20 (p = 1.21e-6)**	**F: 2.93 (±1.96)**	**F: 0.2095**
braingraph	T: 0.16 (p = 7.98e-05)	T: 3.07 (±1.99)	T: 0.2049
86 ROIs	**M: 0.17 (p = 0.0003)**	**M: 3.02 (±2.08)**	**M: 0.2011**
	F: 0.14 (p = 0.0010)	F: 3.03 (±2.01)	F: 0.2163
braingraph	T: 0.16 (p = 2.34e-7)	T: 3.09 (±2.02)	T: 0.2058
129 ROIs	M: 0.13 (p = 0.0031)	M: 3.07 (±2.16)	M: 0.2049
	F: 0.13 (p = 0.0013)	F: 3.04 (±2.07)	F: 0.2171
braingraph	T: 0.17 (p = 4.50e-8)	T: 3.09 (±2.05)	T: 0.2062
234 ROIs	M: 0.14 (p = 0.0025)	M: 3.11 (±2.18)	M: 0.2073
	F: 0.14 (p = 0.0010)	F: 3.05 (±2.14)	F: 0.2179
braingraph	T: 0.17 (p = 1.80e-8)	T: 3.11 (±2.09)	T: 0.2076
463 ROIs	M: 0.15 (p = 0.0005)	M: 3.07 (±2.18)	M: 0.2050
	F: 0.14 (p = 0.0006)	F: 3.06 (±2.18)	F: 0.2183
braingraph	T: 0.19 (p = 1.12e-9)	T: 3.13 (±2.12)	T: 0.2086
1015 ROIs	M: 0.15 (p = 0.0007)	M: 3.06 (±2.14)	M: 0.2038
	F: 0.16 (p = 0.0002)	F: 3.02 (±2.14)	F: 0.2160

Shown is the Pearson correlation *r* between the predicted and actual subjects’ value and its p-value, the mean absolute error (MAE) obtained from a 10-fold cross-validation as well as the range-normalized MAE (NMAE). The highest observed correlation and lowest MAE for each group is highlighted in bold. T: All subjects (total), M: Male subjects only, F: Female subjects only.

**Fig 3:**
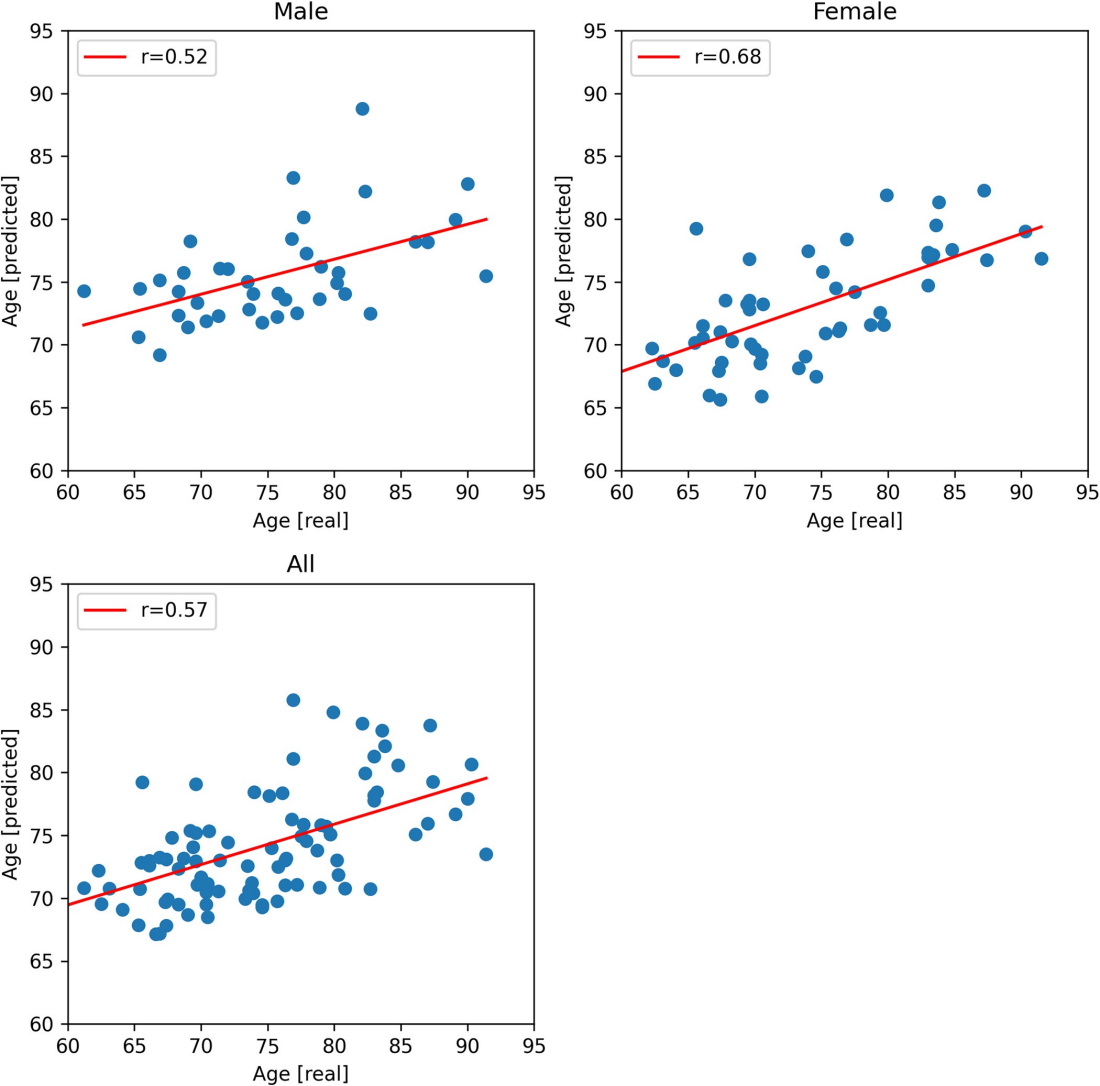
Prediction results for the ADNI dataset (age) for the total dataset (bottom) and the gender-specific subsets (top). The x-axis shows the actual age in years and the y-axis the predicted age in years during the cross-validation.

The prediction result for the ADNI data (Fig 3) achieved a higher correlation value between predicted and real values (r = 0.57) compared to the HCP datasets (r_max_ = 0.22) and a lower NMAE (0.14) compared to the best value out of the HCP datasets (0.20).

Dividing the data into gender-specific groups slightly improved the prediction quality for the ADNI data in case of females (r_F_ = 0.68, NMAE_F_ = 0.14), but slightly decreased it for the male-only subjects (r_M_ = 0.52, NMAE_M_ = 0.17; Table 4). For the HCP data, gender-specific analysis led to inconsistent results and mostly worse prediction outcome when compared to the total dataset, but the female subgroup tended to perform better than the male subgroup.

**Table 4:** Prediction results for the ADNI dataset.

	Pearson‘s *r* (actual vs. predicted values)	MAE	NMAE
All subjects	r = 0.5701 (p = 4.48E-09)	5.05 (±3.78)	0.1448
Male	r = 0.5159 (p = 0.0007)	5.02(±3.54)	0.1662
Female	r = 0.6828 (p = 1.28E-08)	5.07 (±3.43)	0.1449

Prediction results for the age in years based on a 10-fold cross-validation for 94 control subjects from the ADNI dataset (40 males, 54 females).

### Intelligence prediction

The maximal observed Pearson correlation coefficients between the features and intelligence per dataset ranged from 0.127 (129 node braingraph dataset) to 0.168 (1015 ROI braingraph dataset) for total intelligence, from 0.108 (86 ROI braingraph dataset) to 0.139 (1015 ROI braingraph dataset) for fluid intelligence, and from 0.142 (86 ROI braingraph dataset) to 0.189 (1015 ROI braingraph dataset) for crystallized intelligence (S3 Table), The minimal observed Pearson correlation coefficients between the features and intelligence per dataset ranged from -0.153 (86 ROI braingraph dataset) to -0.235 (in-house pipeline) for total intelligence, from -0.129 (86 ROI braingraph dataset) to -0.185 (in-house pipeline) for fluid intelligence, and from -0.151 (129 ROI braingraph dataset) to -0.236 (NICARA) for crystallized intelligence (S4 Table).

The prediction for the different intelligence measures performed similarly between all available datasets. Data from the in-house pipeline showed the lowest MAE for total (11.47) and fluid (9.36) intelligence while the 129 ROI braingraph dataset had the lowest MAE (7.67) in case of crystallized intelligence (Tables 5–7).

**Table 5:** Prediction outcome using the correlation-based regression method for total intelligence.

	Pearson‘s r	MAE	NMAE
NICARA	T: 0.21 (p = 9.97e-12)	**T: 11.47 (±8.29)**	**T: 0.1769**
379 ROIs	M: 0.13 (p = 0.0047)	**M: 11.88 (±8.34)**	**M: 0.1832**
	**F: 0.26 (p = 8.33e-10)**	**F: 11.14 (±8.18)**	**F: 0.1730**
braingraph	T: 0.20 (p = 4.07e-11)	T: 11.59 (±8.26)	T: 0.1787
86 ROIs	M: 0.11 (p = 0.0134)	M: 12.22 (±8.78)	M: 0.1884
	F: 0.19 (p = 9.00e-6)	F: 11.37 (±8.41)	F: 0.1765
braingraph	T: 0.20 (p = 2.88e-11)	T: 11.60 (±8.27)	T: 0.1789
129 ROIs	M: 0.13 (p = 0.0034)	M: 12.20 (±8.86)	M: 0.1880
	F: 0.18 (p = 1.20e-5)	F: 11.44 (±8.41)	F: 0.1777
braingraph	T: 0.21 (p = 1.02e-11)	T: 11.64 (±8.27)	T: 0.1795
234 ROIs	M: 0.14 (p = 0.0022)	M: 12.30 (±8.99)	M: 0.1897
	F: 0.20 (p = 2.71e-6)	F: 11.51 (±8.40)	F: 0.1786
braingraph	T: 0.22 (p = 9.84e-13)	T: 11.68 (±8.27)	T: 0.1801
463 ROIs	**M: 0.16 (p = 0.0005)**	M: 12.36 (±8.80)	M: 0.1906
	F: 0.21 (p = 7.17e-7)	F: 11.58 (±8.41)	F: 0.1798
braingraph	**T: 0.23 (p = 5.67e-14)**	T: 11.73 (±8.34)	T: 0.1808
1015 ROIs	**M: 0.16 (p = 0.0004)**	M: 12.28 (±8.66)	M: 0.1894
	F: 0.22 (p = 1.42e-7)	F: 11.67 (±8.45)	F: 0.1811

Shown is the Pearson correlation *r* between the predicted and actual subjects’ value and its p-value, the mean absolute error (MAE) obtained from a 10-fold cross-validation as well as the range-normalized MAE (NMAE). The highest observed correlation and lowest MAE within each group is highlighted in bold. T: All subjects (total), M: Male subjects only, F: Female subjects only.

**Table 6:** Prediction outcome using the correlation-based regression method for crystallized intelligence.

	Pearson‘s r	MAE	NMAE
NICARA	T: 0.21 (p = 9.93e-12)	T: 7.68 (±5.76)	T: 0.1220
379 ROIs	M: 0.13 (p = 0.0058)	M: 7.94 (±6.07)	M: 0.1312
	**F: 0.25 (p = 1.71e-9)**	**F: 7.43 (±5.49)**	**F: 0.1363**
braingraph	T: 0.21 (p = 2.23e-12)	T: 7.68 (±5.79)	T: 0.1219
86 ROIs	M: 0.16 (p = 0.0005)	M: 7.87 (±6.27)	M: 0.1301
	F: 0.20 (p = 2.38e-6)	F: 7.65 (±5.51)	F: 0.1403
braingraph	T: 0.22 (p = 6.30e-13)	**T: 7.67 (±5.80)**	**T: 0.1218**
129 ROIs	M: 0.18 (p = 5.32e-5)	**M: 7.82 (±6.29)**	**M: 0.1293**
	F: 0.20 (p = 3.19e-6)	F: 7.69 (±5.52)	F: 0.1409
braingraph	T: 0.22 (p = 1.57e-13)	T: 7.69 (±5.79)	T: 0.1221
234 ROIs	M: 0.18 (p = 0.0001)	M: 7.91 (±6.41)	M: 0.1307
	F: 0.21 (p = 4.60e-7)	F: 7.74 (±5.48)	F: 0.1419
braingraph	T: 0.23 (p = 7.58e-14)	T: 7.71 (±5.83)	T: 0.1224
463 ROIs	**M: 0.19 (p = 4.40e-5)**	M: 7.97 (±6.44)	M: 0.1318
	F: 0.22 (p = 1.73e-7)	F: 7.77 (±5.54)	F: 0.1423
braingraph	**T: 0.24 (p = 4.82e-15)**	T: 7.76 (±5.85)	T: 0.1232
1015 ROIs	**M: 0.19 (p = 2.81e-5)**	M: 7.98 (±6.40)	M: 0.1319
	F: 0.23 (p = 7.02e-8)	F: 7.81 (±5.60)	F: 0.1432

Shown is the Pearson correlation *r* between the predicted and actual subjects’ value and its p-value, the mean absolute error (MAE) obtained from a 10-fold cross-validation as well as the range-normalized MAE (NMAE). The highest observed correlation and lowest MAE within each group is highlighted in bold. T: All subjects (total), M: Male subjects only, F: Female subjects only.

**Table 7:** Prediction outcome using the correlation-based regression method for fluid intelligence.

	Pearson‘s r	MAE	NMAE
NICARA	T: 0.14 (p = 3.02e-6)	**T: 9.36 (±6.56)**	**T: 0.1600**
379 ROIs	**M: 0.10 (p = 0.0218)**	**M: 9.76 (±6.82)**	**M: 0.1669**
	**F: 0.21 (p = 6.50e-7)**	**F: 9.07 (±6.28)**	**F: 0.1651**
braingraph	T: 0.13 (p = 2.74e-5)	T: 9.49 (±6.62)	T: 0.1623
86 ROIs	M: 0.04 (p = 0.3265)	M: 10.16 (±7.68)	M: 0.1737
	F: 0.13 (p = 0.0016)	F: 9.25 (±6.42)	F: 0.1683
braingraph	T: 0.13 (p = 2.70e-5)	T: 9.49 (±6.70)	T: 0.1622
129 ROIs	M: 0.02 (p = 0.6377)	M: 10.38 (±7.87)	M: 0.1775
	F: 0.14 (p = 0.0012)	F: 9.29 (±6.46)	F: 0.1691
braingraph	T: 0.13 (p = 3.48e-5)	T: 9.55 (±6.76)	T: 0.1632
234 ROIs	M: 0.02 (p = 0.7231)	M: 10.50 (±8.04)	M: 0.1795
	F: 0.14 (p = 0.0008)	F: 9.35 (±6.53)	F: 0.1702
braingraph	T: 0.14 (p = 3.01e-6)	T: 9.59 (±6.80)	T: 0.1640
463 ROIs	M: 0.06 (p = 0.2238)	M: 10.27 (±7.85)	M: 0.1756
	F: 0.15 (p = 0.0003)	F: 9.43 (±6.58)	F: 0.1717
braingraph	**T: 0.16 (p = 4.46e-7)**	T: 9.67 (±6.89)	T: 0.1653
1015 ROIs	M: 0.07 (p = 0.1515)	M: 10.07 (±7.64)	M: 0.1721
	F: 0.17 (p = 5.68e-5)	F: 9.53 (±6.60)	F: 0.1735

Shown is the Pearson correlation *r* between the predicted and actual subjects’ value and its p-value, the mean absolute error (MAE) obtained from a 10-fold cross-validation as well as the range-normalized MAE (NMAE). The highest observed correlation and lowest MAE within each group is highlighted in bold. T: All subjects (total), M: Male subjects only, F: Female subjects only.

**Fig 4:**
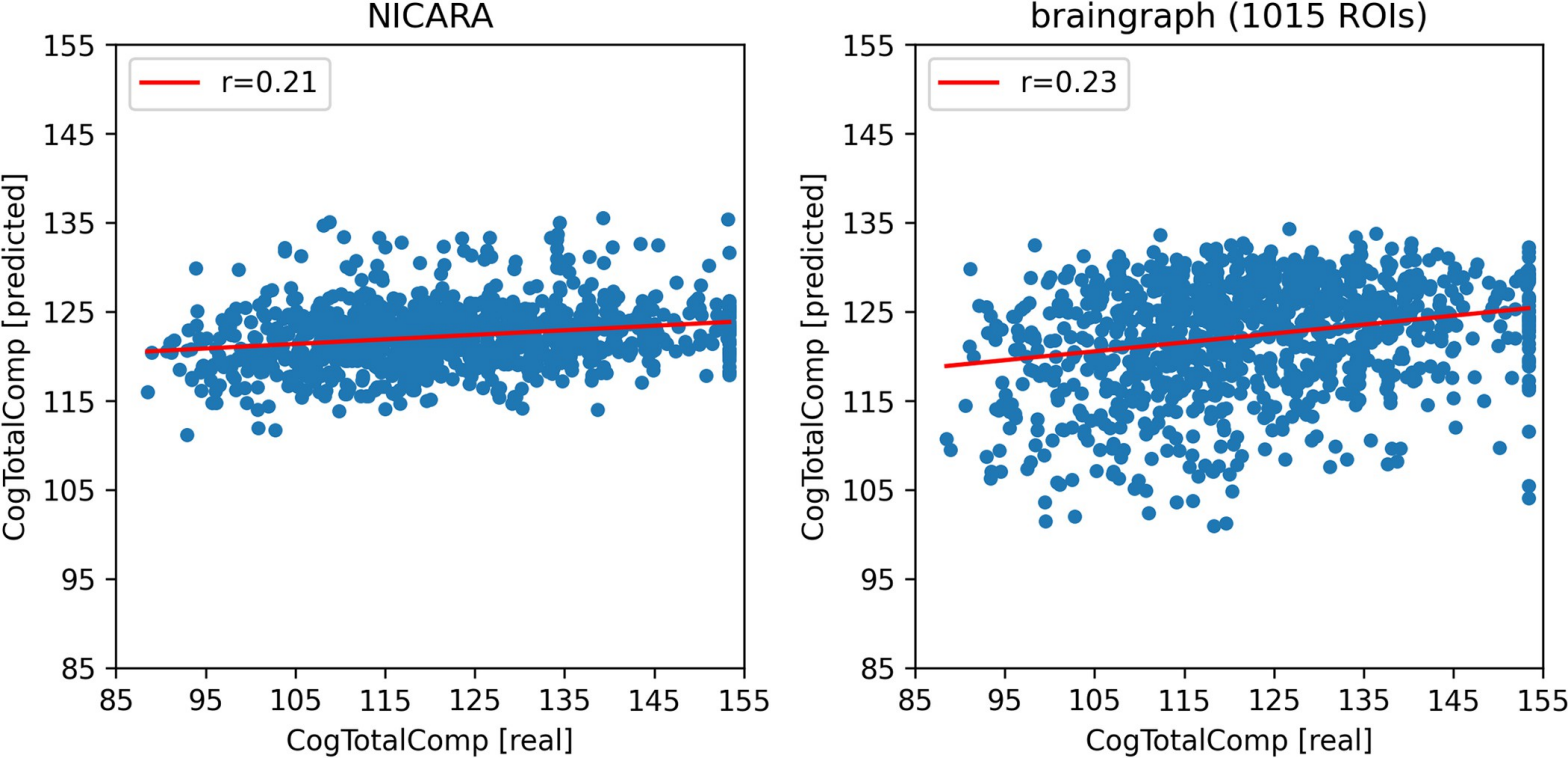
Prediction results for the HCP dataset (total intelligence) for NICARA and the 1015 ROI dataset from braingraph.org. The x-axis shows the actual values and the y-axis the predicted values during the cross-validation.

**Fig 5:**
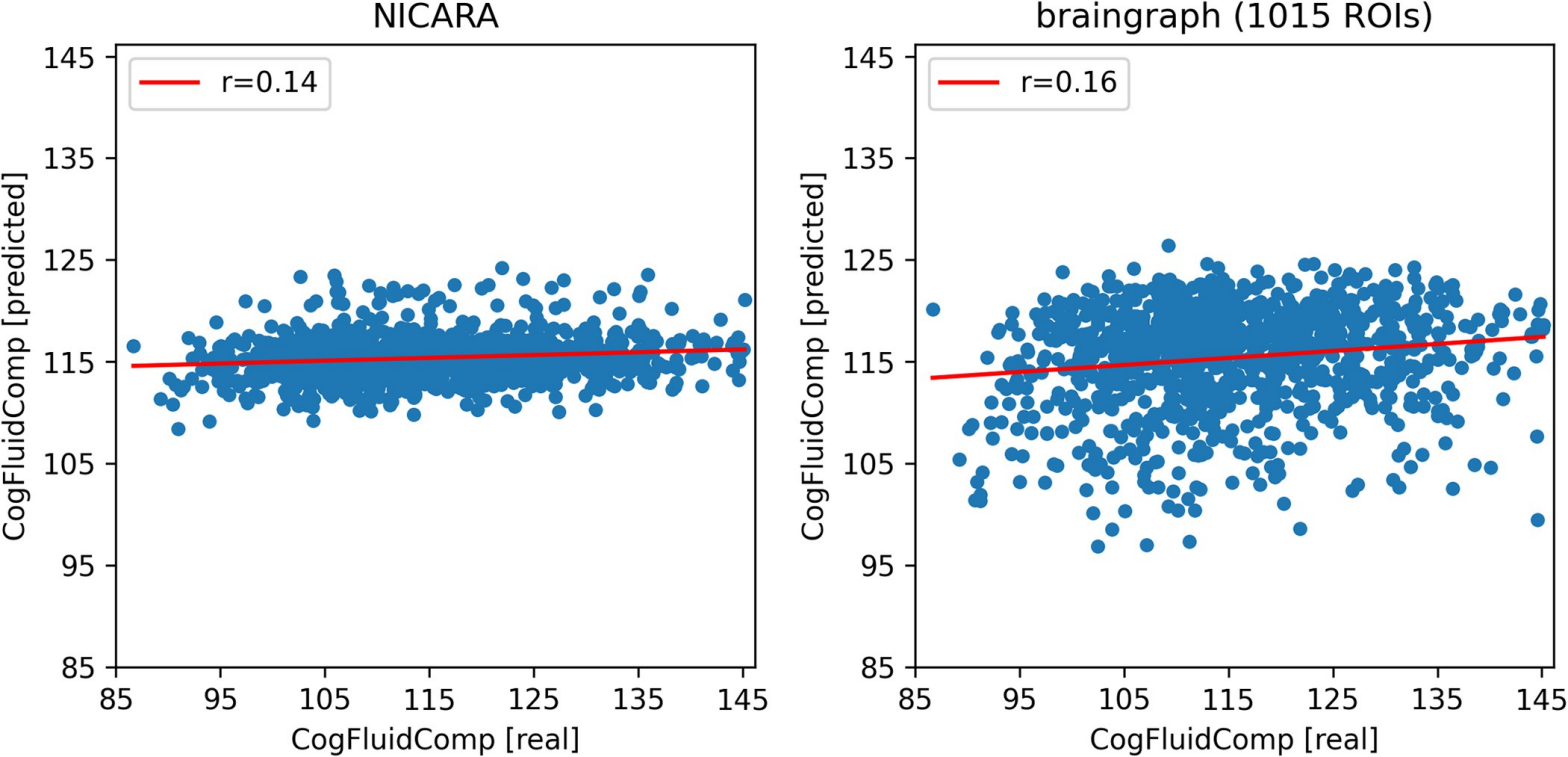
Prediction results for the HCP dataset (fluid intelligence) for NICARA and the 1015 ROI dataset from braingraph.org. The x-axis shows the actual values and the y-axis the predicted values during the cross-validation.

**Fig 6:**
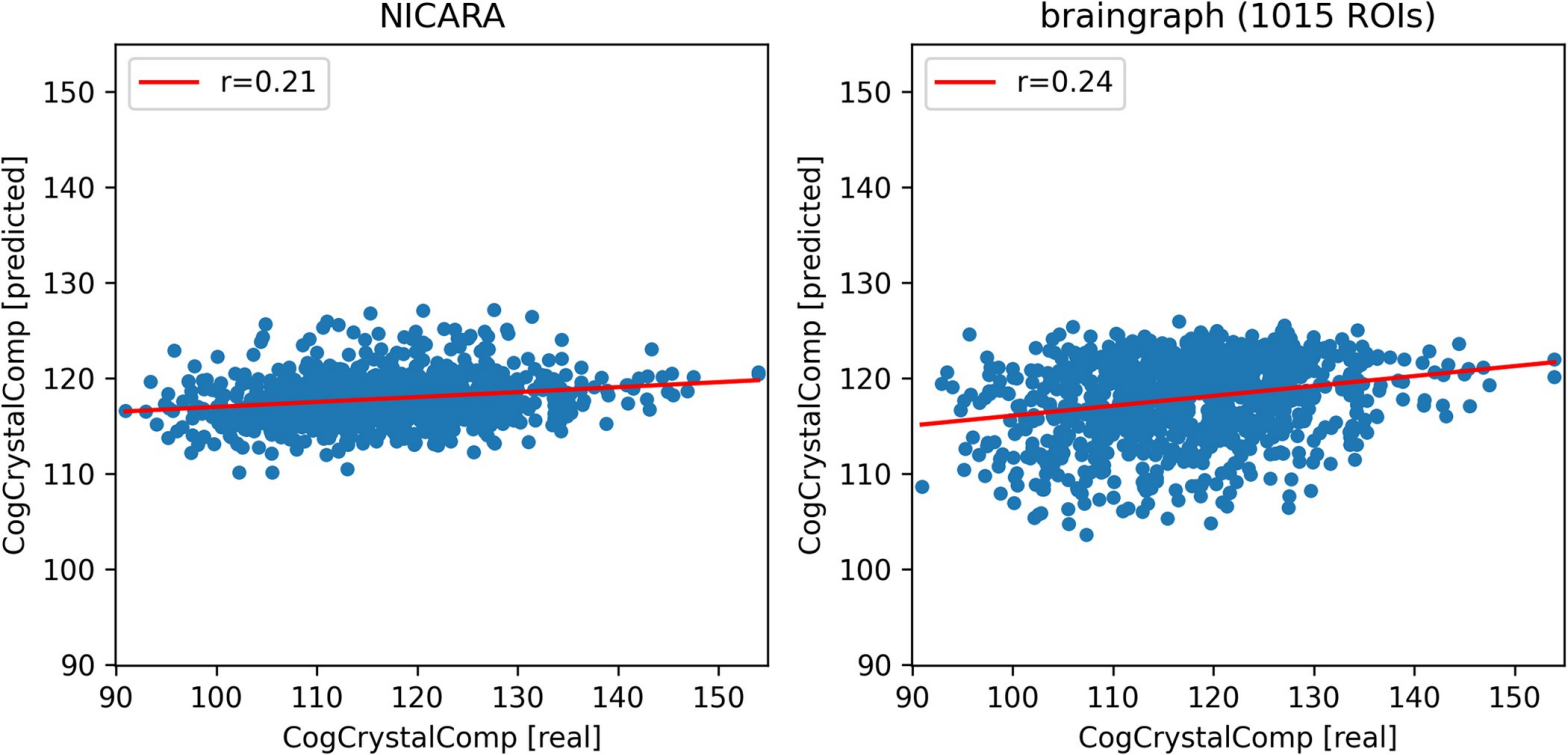
Prediction results for the HCP dataset (crystallized intelligence) for NICARA and the 1015 ROI dataset from braingraph.org. The x-axis shows the actual values and the y-axis the predicted values during the cross-validation.

The braingraph dataset with 1015 ROIs showed the highest correlation values for all intelligence measures for all subjects with r = 0.24 (Tables 5–7; Figs 4–6). Within the braingraph datasets, increasing ROI number showed the tendency to increase the MAE but also slightly the correlation between actual and predicted values. For total intelligence, only the absolute error differences between the NICARA and 1015 ROI braingraph dataset was significant based on the Wilcoxon signed-rank test (p = 0.0411). For fluid intelligence, there were only significant differences for 234 vs. 1015 ROI braingraph (p = 0.0341) and 463 vs 1015 ROI braingraph (p = 0.0140). Lastly, for crystallized intelligence, several absolute error differences were significant: NICARA vs. 463 ROI braingraph (p = 0.0489), NICARA vs. 1015 ROI braingraph (p = 0.0195), 86 vs 1015 ROI braingraph (p = 0.0280), 129 vs 234 ROI braingraph (p = 0.0399), 129 vs. 1015 ROI braingraph (p = 0.0125), 234 vs 1015 ROI braingraph (p = 0.0238), and 463 vs. 1015 ROI braingraph (p = 0.0273). None of the p-values remained significant after applying the Benjamini-Hochberg multiple testing correction (FDR = 0.05).

For the comparisons of absolute errors in the gender-specific subgroups for total intelligence, 129 vs 234 ROI braingraph (p = 0.045) was significant in the male subgroup and 86 vs 463 ROI (p = 0.0362), 86 vs 1015 ROI (p = 0.0128), 129 vs 1015 ROI (p = 0.0223), and 234 vs 1015 ROI (p = 0.0231) in the female subgroup before but not after multiple testing correction. For crystallized intelligence, 129 vs 234 ROI (p = 0.0417) showed significance in the male subgroup and the following in the female subgroup: NICARA vs 234 ROI (p = 0.0253), NICARA vs 463 ROI (p = 0.0252), and NICARA vs 1015 ROI (p = 0.0262). None of the p-values remained significant after multiple testing correction. For fluid intelligence in the male subgroup, the following comparisons were significant before multiple testing correction: NICARA vs 234 ROI (p = 0.0285), 86 vs 129 ROI (p = 0.0357), 86 vs 234 ROI (p = 0.0178), 129 vs 1015 ROI (p = 0.0140), 234 vs 463 ROI (p = 0.0107), 234 vs 1015 ROI (p = 0.0010), 463 vs 1015 ROI (p = 0.0332). The comparison between 234 and 1015 ROI braingraph datasets did remain significant even after the multiple testing correction. In the female subgroup for fluid intelligence, the following comparisons were significant before but not after multiple testing correction: 86 vs 463 ROI (p = 0.0227), 86 vs 1015 ROI (p = 0.0058), 129 vs 463 ROI (p = 0.0376), 129 vs 1015 ROI (p = 0.0110), 234 vs 463 ROI (p = 0.0496), 234 vs 1015 ROI (p = 0.0086), and 463 vs 1015 ROI (p = 0.0313).

**Fig 7:**
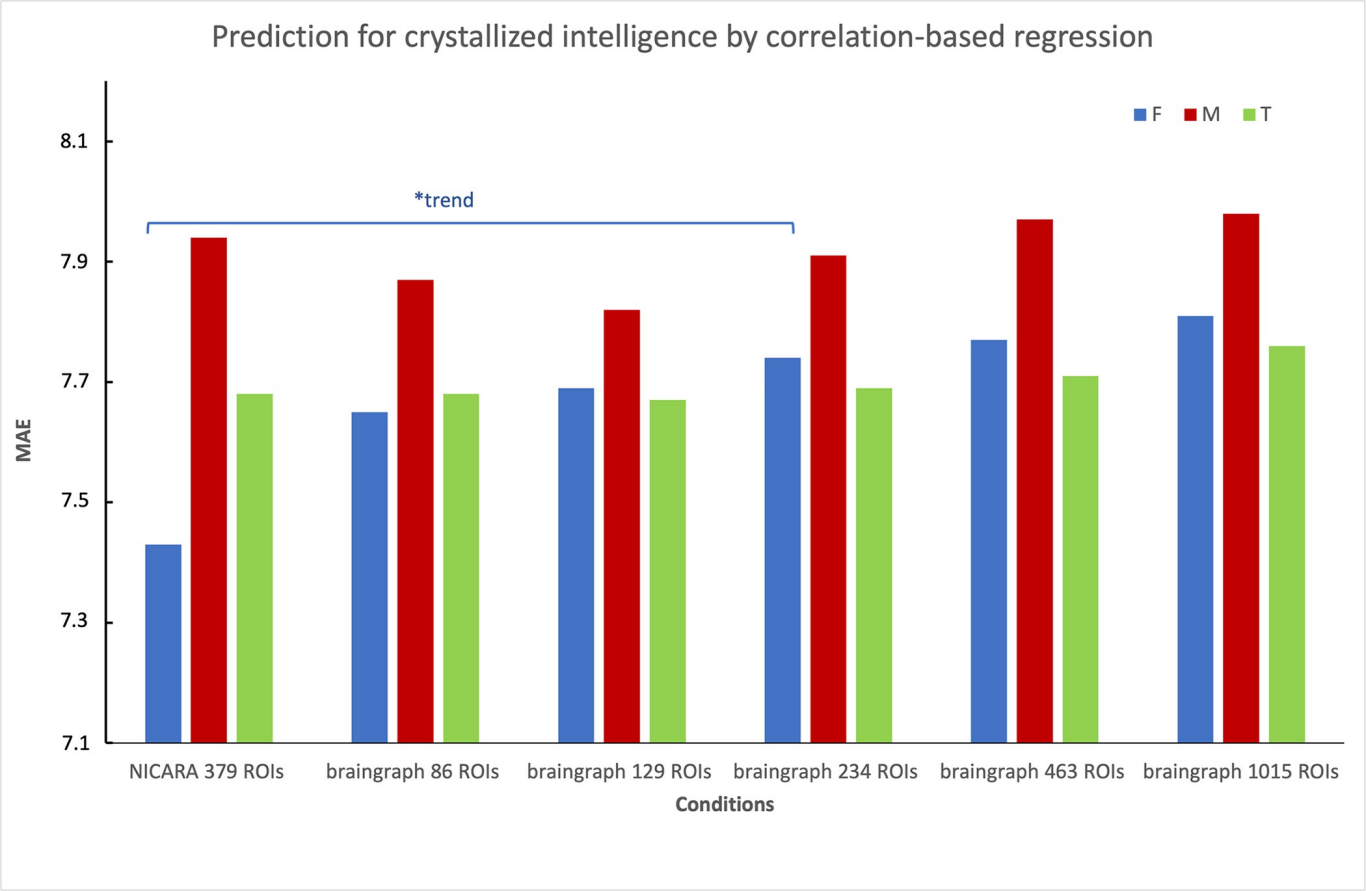
MAE of crystallized intelligence prediction. Total dataset: green, Female subset: blue, Male subset: red. *: p < 0.05 without correction based on the Wilcoxon signed-rank test for paired absolute errors.

**Fig 8:**
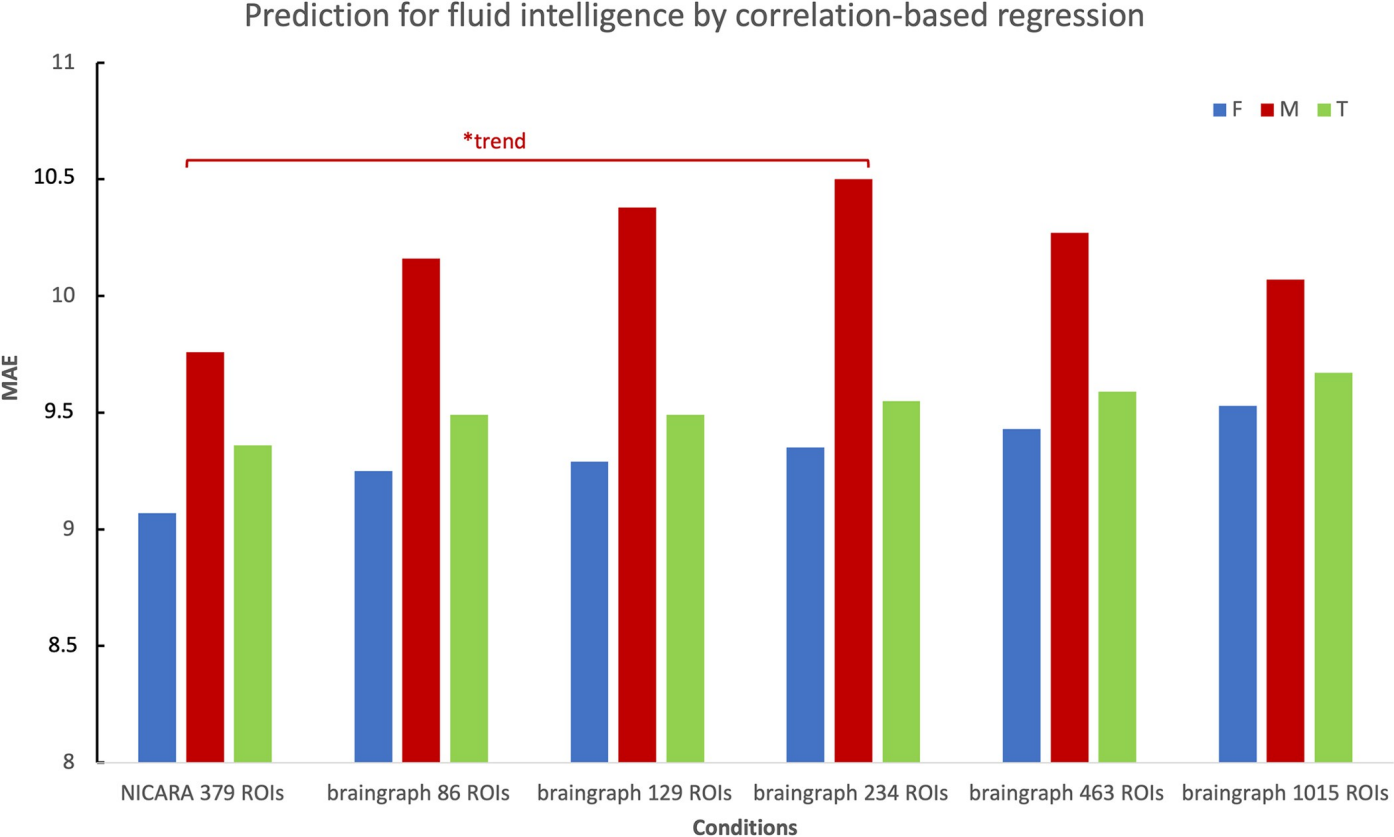
MAE of fluid intelligence prediction. Total dataset: green, Female subset: blue, Male subset: red. *: p < 0.05 without correction based on the Wilcoxon signed-rank test for paired absolute errors.

Considering the relatively good Pearson correlation scores of NICARA among gender-specific groups versus pipeline conditions for both crystallized and fluid intelligence, we report two interesting trends from the gender-specific results by Wilcoxon signed-rank test of the absolute errors: The first trend shows difference in female group between NICARA and 234 ROI braingraph dataset for crystallized intelligence (p = 0.025; Fig 7). And the second trend reveals a difference in the male group for same comparisons of fluid intelligence (p = 0.0285; Fig 8).

Since the cognition fluid and crystallized composite score have the same range and we also applied the Wilcoxon signed-rank test to the absolute error differences between them. The differences were significant for all datasets (Table 8) and all of them remained significant after a Benjamini-Hochberg multiple testing correction (FDR = 0.05).

**Table 8:** P-values from the comparison between crystallized and fluid intelligence.

	NICARA	braingraph	braingraph	braingraph	braingraph	braingraph
	379 ROIs	86 ROIs	129 ROIs	234 ROIs	463 ROIs	1015 ROIs
Total	1.38e-10	4.04e-11	4.19e-11	2.18e-11	2.62e-11	1.84e-11
Male	3.26e-06	8.30e-07	7.12e-08	7.35e-08	2.86e-06	1.65e-05
Female	2.18e-06	6.24e-06	6.42e-06	6.33e-06	2.26e-06	8.32e-07

P-values from the Wilcoxon signed-rank test between the absolute errors from crystallized and fluid intelligence prediction for each dataset. All p-values remained significant after the Benjamini-Hochberg multiple testing correction (FDR = 0.05).

Gender-specific analysis led to a worse prediction outcome for males in case of total intelligence for all datasets compared to the results for all subjects. For females, the outcome improved for all datasets except for the 1015 ROI dataset based on the NMAE, but the correlation value only improved for the NICARA dataset compared to the total data. For crystallized and fluid intelligence, gender-specific analysis did not improve the outcome. In case of fluid intelligence, the correlation values between actual and predicted values for the male subgroup in the braingraph datasets were not even significant anymore (Table 7).

## Discussion

In this study, we investigated the predictability of age and intelligence measures based on whole brain structural connectome measurement under various conditions of imaging processing and gender. First, we demonstrated that NICARA processing combined with our machine learning approach could well predict age of ADNI subjects but performed worse for HCP subjects consistent with braingraph.org results. Second, crystallized intelligence was better predicted than fluid intelligence in general. Finally, we found two interesting trends of gender effects for fluid and crystallized intelligence predictability that did not remain significant after multiple testing correction.

Using a simple machine learning approach based on whole brain structural connectivity only we were able to decently predict age for cognitively normal ADNI-3 control subjects (N = 94) with a distinct age range (r = 0.57, NMAE = 0.14). Our finding is similar to that of recent studies both in MAE and std of age prediction using structural connectivity data of ADNI [36, 37], although both studies employed different workflows to extract structural connectivity features and different prediction methods.

However, the prediction quality for age was worse in case of the HCP young adult dataset (r = 0.21, NMAE = 0.20), which only covered a narrow range of young subjects, for both investigated pipelines (in-house and braingraph.org) under different parcellation methods despite its large size (N = 1048). Multi-factor characteristics of HCP Young Adult dataset structural connectivity could account for limited predictability of age. This effect of the HCP dataset is evident by comparable age prediction quality from distinct processing approaches of NICARA versus braingraph.org.

For subjects of the HCP Young Adult study, total, fluid and crystallized intelligence values were measured as cognition composite scores. The overall low predictability of intelligence by whole brain structural connectome features confirmed the statement of Wu and colleagues’ work [38] that their prediction combining cortical and subcortical surfaces together yielded the highest accuracy of fluid intelligence for both ABCD (N = 8070, r = 0.314) and HCP datasets (N = 1097, r = 0.454), outperforming the state-of-the-art prediction of fluid intelligence from any other brain measures in the literature. Wu and colleagues developed a novel graph convolutional neural networks (gCNNs) for the analysis of localized anatomic shape and prediction of fluid intelligence. Krämer et al. [39] reported a similar outcome to this study for a small trend for a multimodal benefit therefore concluded that developing a biomarker for cognitive aging remained challenging. Their study employed multimodal information, i.e., region-wise grey matter volume (GMV), resting-state functional connectivity (RSFC), and structural connectivity (SC), and generalized results across different ML approaches in 594 healthy older adults (age range: 55–85 years) from the 1000BRAINS dataset.

Predictability of crystallized intelligence was better than that of fluid intelligence in all investigated datasets based on the Wilcoxon signed-rank test applied to the paired absolute errors (Table 8), which may imply a stronger relation between crystallized intelligence and whole brain white matter probabilistic tractography based connectivity than that of fluid intelligence. Similar findings have been published by investigating distinct multi-region neuroanatomical patterns extracted from grey matter surface as well as volumetric assessments in 1089 HCP subjects by employing an elastic net regression model [40]. Our finding is also consistent with another study that investigated 415 HCP subjects by a similar tractography method but using only 86 ROIs from FreeSurfer [41]. A much finer atlas parcellation with 439 ROIs created in that study did not show a significant difference in predictability between crystallized and fluid intelligence [41]. Other papers considered highly correlated neuroanatomical morphometry profiles, i.e. cortical surface area, and the environmental impact on the relevant neuroanatomical morphometry as explanations for better predictability of crystallized intelligence compared to fluid intelligence [40, 42].

Interestingly, we find tendencies of slightly better prediction of intelligence in NICARA by gender-specific subgroup analysis based on the range-normalized mean absolute errors and pearson correlation between actual and predicted values. This supports the finding that gender specific factors [43–48] may affect connectivity as well as the relationship between connectomics and cognition [11].

With the publicly available braingraph.org dataset, connectome data from a second structural connectome extraction method for the same HCP subjects was available with five different parcellations (86, 129, 234, 463 and 1015 ROIs). Within the braingraph datasets, the MAE showed the tendency to increase with a higher ROI number. This may be due to the parcellation method itself, or the fact that the resulting connectivity matrix might be too sparse using only 1 million streamlines for finer parcellation [41, 49–51]. However, the differences between the different braingraph datasets were not significant after correcting the p-values obtained by Wilcoxon signed-rank tests between the paired absolute errors for multiple testing using the Benjamini-Hochberg method (FDR = 0.05).

### Limitations

An obvious shortcoming of the HCP Young Adult subjects for prediction of age and intelligence are the biases in the data distribution. The data only contained young subjects (aged 22–37 years), and the cognition composite scores are clearly biased towards higher values. 100 is the United States average but the mean values for the three scores of the 1048 subjects were all higher than that (Table 1). We could show the influence of the age range by also applying the approach to control subjects from the ADNI-3 study with an age range about twice as large (Δ = 35.0 years vs. Δ = 15 years) resulting in a better prediction performance (reflected by a lower NMAE and a higher correlation between actual and predicted values), even though these subjects were also biased as they contained elderly individuals only (56.5–91.5 years).

Apart from the bias, a possible explanation for the low predictability of intelligence in this study can be seen in the structural correlates of intelligent behavior. Human intelligence is thought to be associated with physiological and morphological properties of cortical pyramidal neurons [52], which, of course, can only be captured indirectly by whole brain DTI-based fiber tracking.

### Future approaches

To certify the age prediction, it would be favorable to apply the proposed approach to a dataset with a wider age range such as the Lifespan Human Connectome Project in Aging [53] once it is complete and available. Moreover, it has been previously discussed whether sets of connections would uniquely map onto cognitive function [54]. Further work could elaborate on this idea to test whether specific functional brain networks [55] and their connectedness are more predictive than individual or sets of connections alone. From studies with multiple sclerosis patients, it is known that graph theoretical measures are better descriptors of cognitive decline [56, 57] than the strengths of individual connections. For future studies on prediction qualities of the connectome for cognitive measures it would therefore be favorable to include graph theoretical measures [58] as well. Combining structural and functional brain networks for intelligence prediction revealed so far ambivalent results [40]. Here is certainly more work needed, for example, by using brain multiplex networks [59] to increase predictive power of multi-modal connectomes. Being able to predict brain age and intelligence from brain connectivity has a large impact on monitoring disease progression in dementia or other brain diseases associated with cognitive decline, for example, multiple sclerosis.

### Conclusion

In conclusion, this study explores the predictability of age and intelligence in cognitive normal subjects from the HCP and ADNI datasets by means of whole brain tractography-based structural connectome applying a simple and easy-to-use established machine-learning method. To our knowledge, this is the first study focusing on a single neuroimaging modality feature to predict age and intelligence from whole-brain tractography. The good predictability of age in ADNI and the finding that crystallized intelligence was better predictable than fluid intelligence in HCP datasets was possible by the combination of the NICARA structural connectome pipeline and a simple machine learning model. Therefore, we believe that such a combination could provide a reliable framework option to further narrow down the gap in prediction between neuroimaging features and subjects’ cognition as well as other biological features.

## Supporting information

S1 Table 1048 HCP subject IDs used for the analysis. (XLSX)

S2 Table 94 ADNI subject IDs used for the analysis. (XLSX)

S3 Table Maximal observed Pearson correlation coefficients (HCP datasets). (DOCX)

S4 Table Minimal observed Pearson correlation coefficients (HCP datasets). (DOCX)

S1 File Python3 implementation of the correlation-based regression algorithm. (PY)
