# Supplementary material for: Predictability of intelligence and age from structural connectomes
Source: PLoS One. 2024 Apr 1;19(4):e0301599. doi: 10.1371/journal.pone.0301599 (PMC10984540; doi:10.1371/journal.pone.0301599)
Supplement: S3 Table — (DOCX) [file pone.0301599.s003.docx]

|  | NICARA (379 ROIs) | braingraph (86 ROIs) | braingraph (129 ROIs) | braingraph (234 ROIs) | braingraph (463 ROIs) | braingraph (1015 ROIs) |
| --- | --- | --- | --- | --- | --- | --- |
| Age [years] | **0.227** | 0.114 | 0.105 | 0.131 | 0.128 | 0.126 |
| Intelligence, total | 0.133 | 0.132 | 0.127 | 0.129 | 0.156 | **0.168** |
| Intelligence, fluid | 0.116 | 0.108 | 0.117 | 0.133 | 0.122 | **0.139** |
| Intelligence, crystallized | 0.150 | 0.142 | 0.162 | 0.161 | 0.186 | **0.189** |

**Supplementary Table 3.** Maximal observed Pearson correlation coefficients between the individual connectivity strengths (features) and each investigated attribute in the different HCP datasets (see Eq. 2). Maximal value per attribute (rows) out of all data sets (columns) is displayed in bold.
